# Supplementary material for: PHI-1, an Endogenous Inhibitor Protein for Protein Phosphatase-1 and a Pan-Cancer Marker, Regulates Raf-1 Proteostasis
Source: Biomolecules. 2023 Dec 4;13(12):1741. doi: 10.3390/biom13121741 (PMC10741526; doi:10.3390/biom13121741)
Supplement: Supplementary file 1 [file biomolecules-13-01741-s001.zip › biomolecules-2744835-Supplementary Figure S1.pdf]

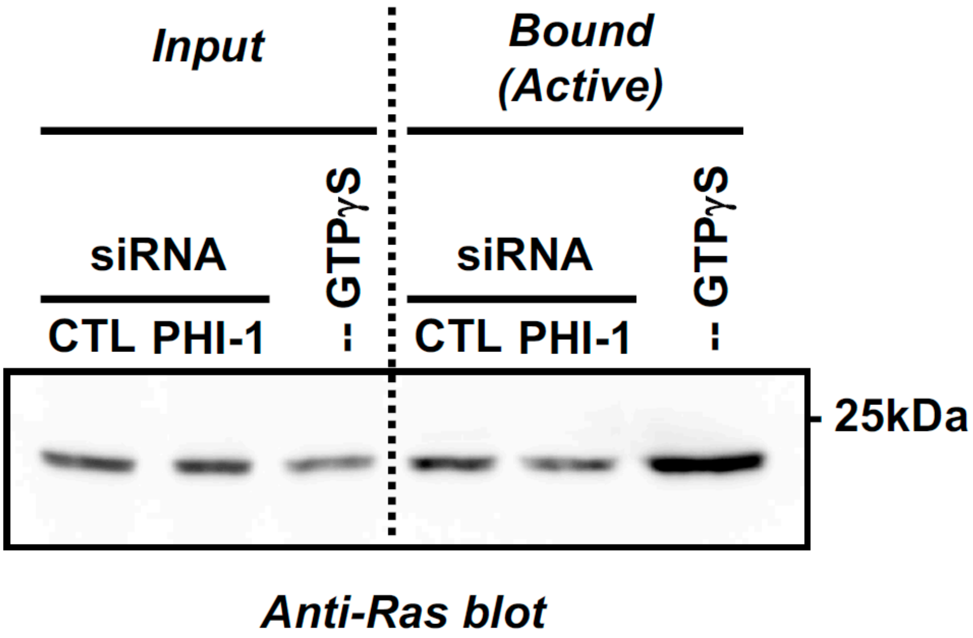

Supplemental Figure S1: Ras-GTP-pulldown assay. HEK293 cells were treated with siRNA for PHI-1 or control (CTL), and then subjected to Ras-bining domain pulldown assay, as described in Stevenson et al., FEBS Lett 578 (2004) 73-79. GTPgS was used for the positive control.
