# Supplementary figures and images for: PHI-1, an Endogenous Inhibitor Protein for Protein Phosphatase-1 and a Pan-Cancer Marker, Regulates Raf-1 Proteostasis
Source: Biomolecules. 2023 Dec 4;13(12):1741. doi: 10.3390/biom13121741 (PMC10741526; doi:10.3390/biom13121741)

Uncropped blot images

Figure 2

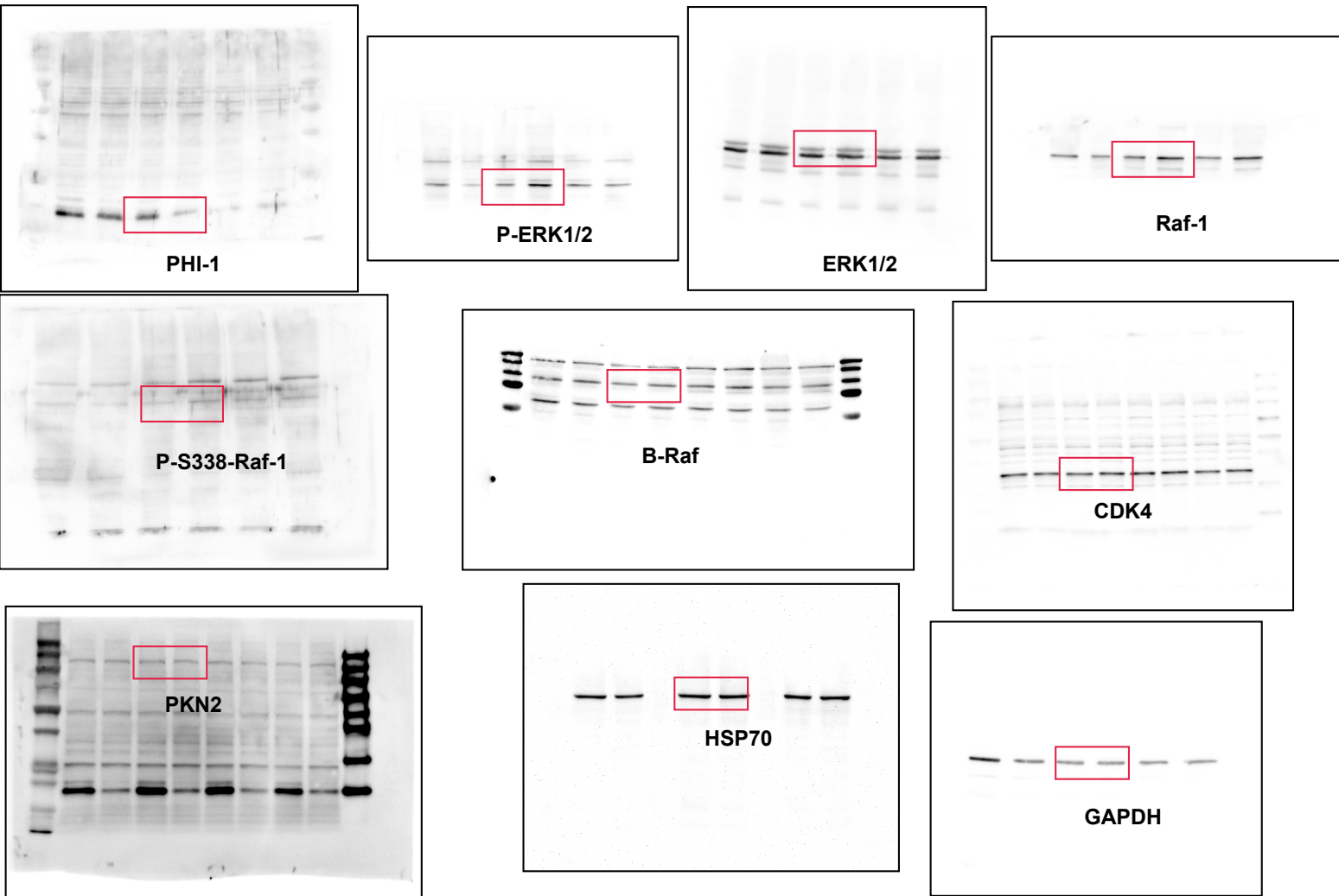

Figure 3A

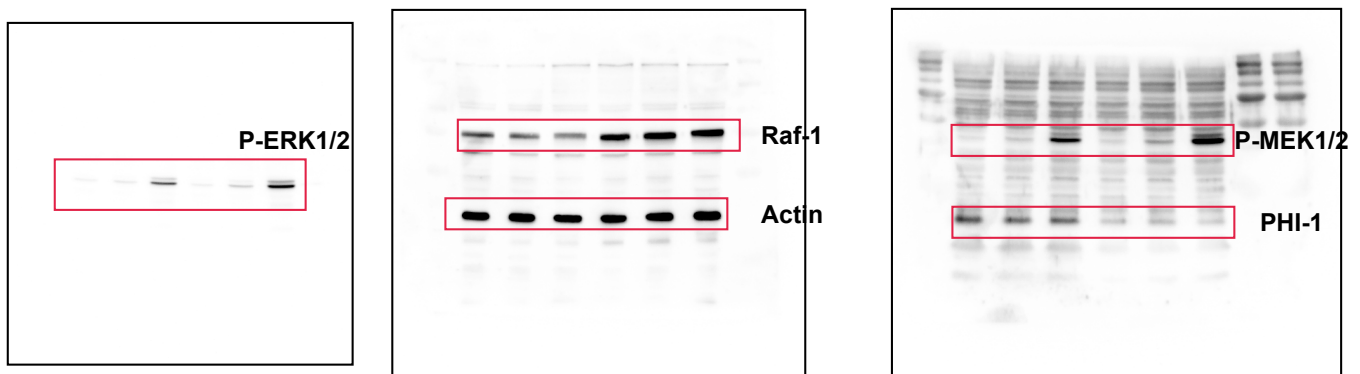

Figure 3C

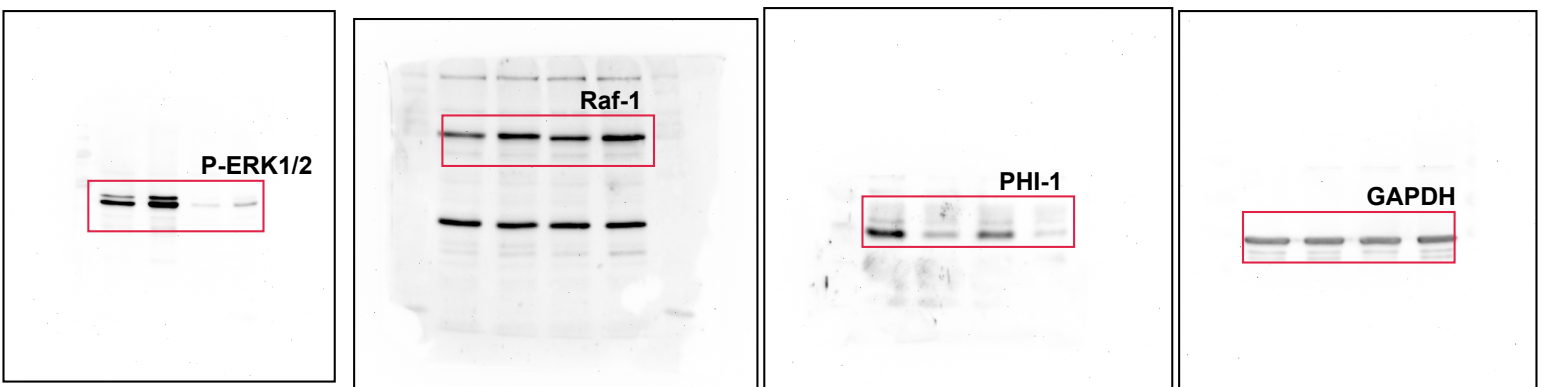

Figure 4A

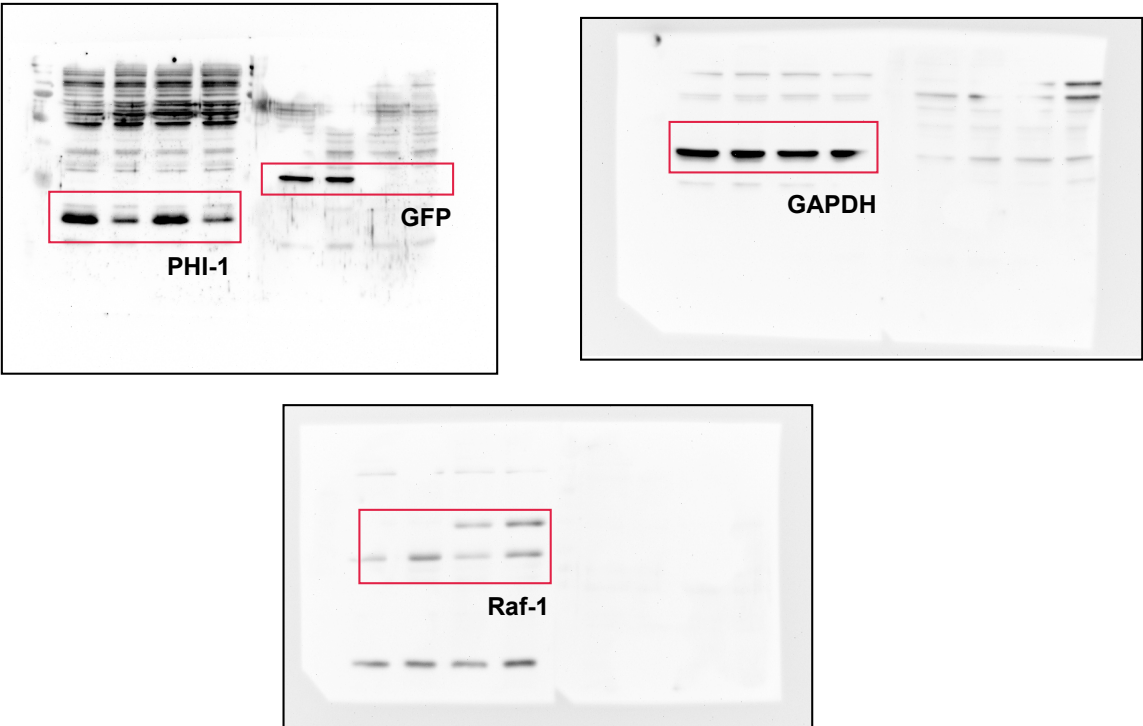

Figure 4B

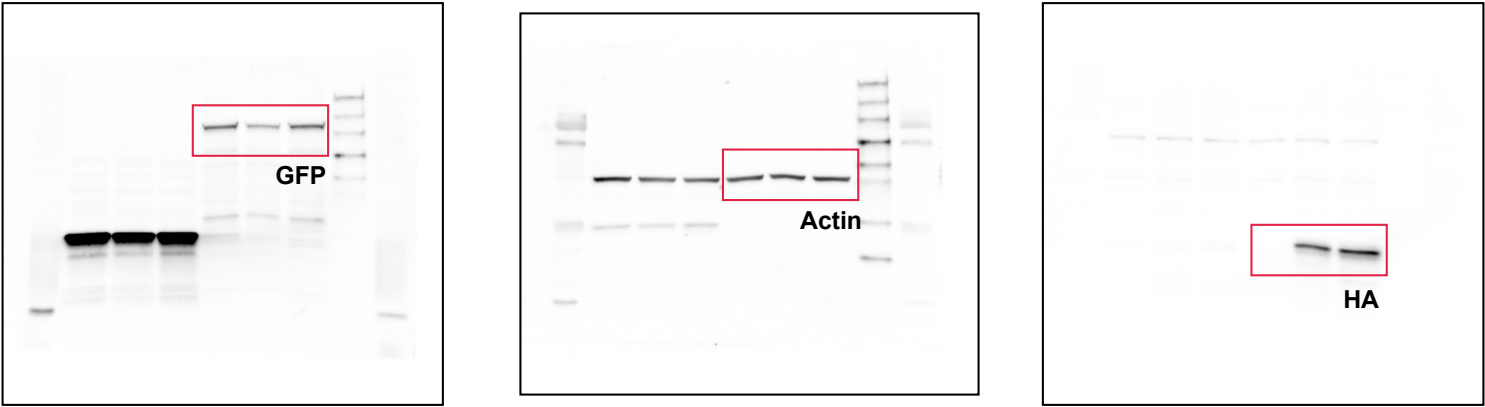

Figure 5

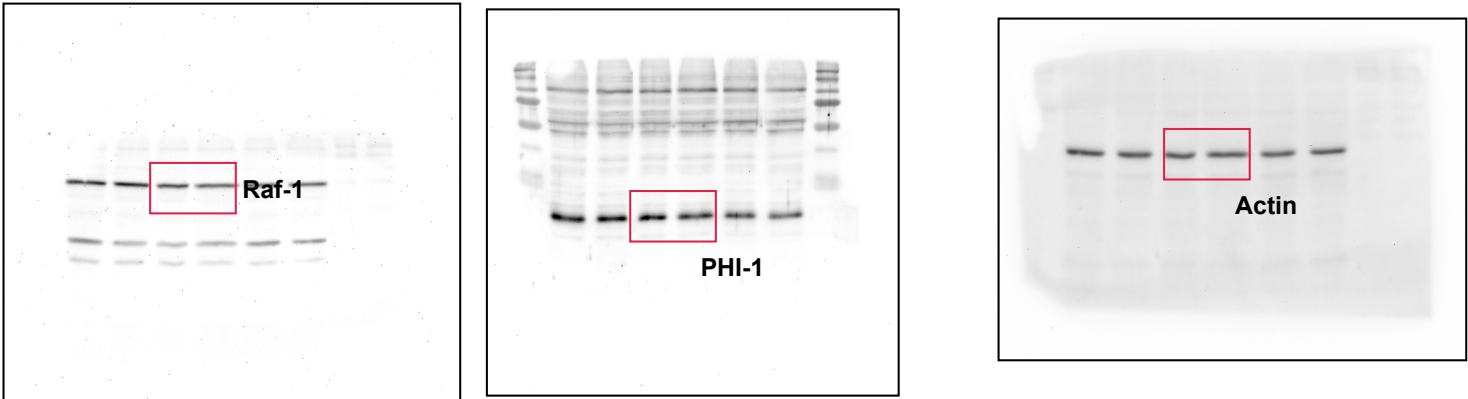

Supplement: Supplementary file 1 [file biomolecules-13-01741-s001.zip › biomolecules-2744835-original-images.pdf]

Uncropped images

Figure 1B

Anti-PHI-1

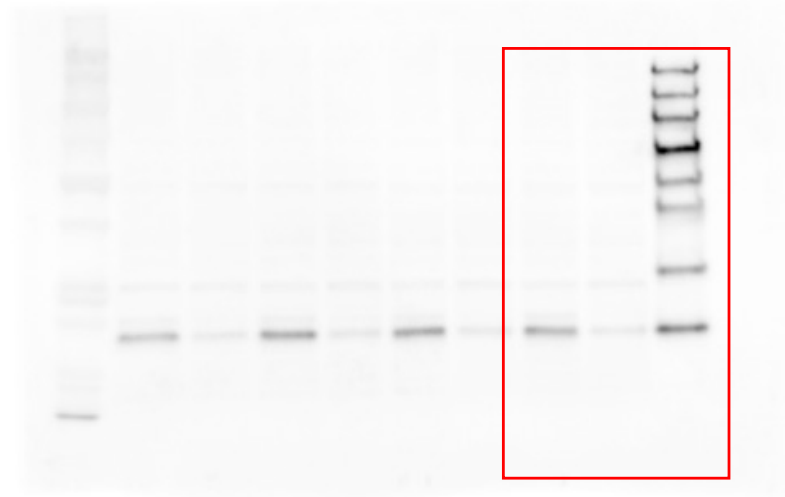

Anti-actin

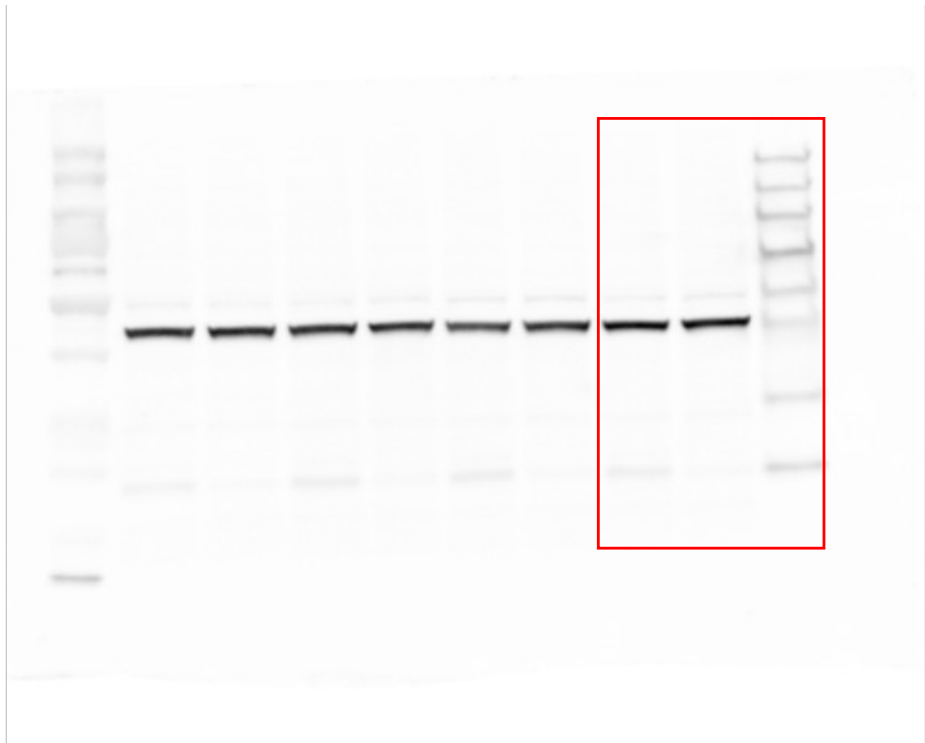

Supplement: Supplementary file 1 [file biomolecules-13-01741-s001.zip › biomolecules-2744835-Uncropped figure 1B.pdf]
